# Impact of diverse learning resources and support system on Chinese English language learners’ motivation in virtual and traditional learning settings

**DOI:** 10.3389/fpsyg.2026.1705007

**Published:** 2026-09-07

**Authors:** Shiqi Xie, Ahmad Albattat

**Affiliations:** 1 School of Foreign Languages, Zhengzhou College of Finance and Economics, Zhengzhou, China; 2 School of Global Hospitality and Tourism, Asia Pacific University of Technology & Innovation, Federal Territory of Kuala Lumpur, Malaysia

**Keywords:** Chinese English language learners, learning results, self-regulated learning, traditional learning settings, virtual learning settings

## Abstract

**Introduction:**

While Self-Determination Theory (SDT) establishes the importance of autonomy, competence, and relatedness for motivation, less is known about how these needs are specifically fueled within digital learning ecosystems. Prior research often examines learning resources, support systems, and delivery modes (virtual vs. traditional) in isolation. This study addresses this gap by positing engagement as the critical mediator that explains how these combined structural and support factors fulfill psychological needs and, in turn, enhance motivation. The novel integrated model tested here uniquely combines SDT with a learning engagement framework to reveal the precise pathways through which educational environments drive motivational outcomes.

**Methods:**

Using a quantitative approach, data were collected from students and analyzed through Structural Equation Modeling (SEM) via Smart-PLS.

**Results:**

The findings indicate significant direct effects, with diverse learning resources (H1: *β* = −0.453, *t* = 5.624, *p* = 0.000) and support systems (H2: *β* = 0.263, *t* = 2.883, *p* = 0.004) directly influencing engagement. Mediation analysis revealed complementary and competitive partial mediation effects, such as support systems enhancing motivation through engagement (H2: *β* = 0.413, *t* = 6.066, *p* = 0.000) and diverse learning resources exhibiting competitive partial mediation (H1: *β* = −0.512, *t* = 7.452, *p* = 0.000). Conversely, learning mode showed no significant mediation effect (H3: *β* = −0.025, *t* = 0.554, *p* = 0.580).

**Discussion:**

These findings reinforce SDT’s premise that intrinsic and extrinsic factors shape student motivation, providing theoretical insights into motivation-driven learning. Practically, the study suggests enhancing support systems and engagement strategies in educational settings. The study contributes to educational psychology by extending SDT into digital and traditional learning contexts, with implications for curriculum design and policy development.

## 1 Introduction

The globalization of English as a lingua franca has significantly influenced language education in non-English-speaking countries, particularly in China, where English proficiency is increasingly essential for academic and professional success (Abro et al., 2025). The rapid advancement of educational technology and digital learning platforms has introduced new opportunities for English language learners, offering diverse learning resources and support systems that were previously unavailable (Matiyenga and Khoalenyane, 2025). However, despite these developments, disparities persist in how learners engage with these resources in virtual and traditional learning settings, affecting their motivation, self-regulation, and overall learning outcomes (Hu and Xiao, 2025). Given China’s ambition to enhance its global competitiveness through improved English proficiency, it is crucial to investigate how different learning modes and support structures contribute to learner motivation and academic performance.

The globalization of English as the world’s primary lingua franca has fundamentally reshaped language education, particularly in nations like China where English proficiency is now a critical determinant of academic and professional success. English’s status as the number one global language is anchored in historical, economic, and technological factors. In this era of globalization, English operates as the essential medium for accessing cutting-edge research, participating in the global economy, and fostering cross-cultural collaboration. Consequently, mastering English is not merely an academic subject in China but a strategic imperative for national competitiveness and individual opportunity. The rapid advancement of educational technology has introduced new avenues for acquiring this vital skill, offering learners diverse digital resources and support systems previously unavailable. However, significant disparities persist in how learners engage with these tools across virtual and traditional settings, directly impacting their motivation, self-regulation, and ultimate learning outcomes. Given China’s explicit ambition to bolster its global standing, investigating how different learning modes and support structures can most effectively cultivate English proficiency is therefore a matter of pressing national and educational importance.

In China, English language education is undergoing a transformative shift as institutions increasingly adopt hybrid learning models integrating both traditional classroom instruction and virtual platforms (Makhija et al., 2025). The implementation of digital resources, including online exercises, multimedia content, and interactive applications, aims to enhance engagement and facilitate autonomous learning (Sabirjonova, 2025). However, studies suggest that students face challenges in maintaining motivation and self-regulation in online learning environments due to limited peer interaction, inconsistent teacher support, and technological constraints (Kulal et al., 2024). Additionally, the effectiveness of these diverse learning resources and support systems varies among learners with different proficiency levels, raising concerns about equity in language learning outcomes (Yingsoon et al., 2025). This study seeks to address these issues by systematically comparing the impact of learning resources, support systems, and learning modes on Chinese English language learners.

In the case of English language learning in China, these generic challenges are critically intensified by a unique pedagogical ecosystem. The prevalent exam-oriented culture prioritizes discrete grammar and vocabulary acquisition over communicative competence, a misalignment with online platforms that could otherwise excel at fostering interaction. More profoundly, the traditional learning model fosters a high reliance on teacher authority for direction, corrective feedback, and motivational oversight. When shifted to a virtual setting with reduced synchronous contact and less immediate pedagogical support, this reliance creates a support vacuum. The consequence is not merely logistical inconvenience; it directly undermines the psychological needs outlined in Self-Determination Theory.

One of the primary concerns in the English language education sector in China is the disparity in learning outcomes between students in virtual and traditional settings (Zhu et al., 2025). Traditional classroom-based learning offers direct teacher guidance, structured peer interaction, and immediate feedback, which are essential for language acquisition (Hamdayani and Sapitri, 2025). Conversely, virtual learning environments provide flexibility and accessibility but may lack sufficient support mechanisms, leading to disengagement and lower self-regulation among students (Yorganci, 2025). The lack of empirical studies examining how these contrasting environments affect learners’ motivation, self-regulation, and academic performance highlights the need for further research in this area (Stavropoulou et al., 2025).

While challenges like limited interaction are well-documented in online learning broadly, their impact is critically mediated by China’s specific educational context. The pervasive exam-oriented culture filters all learning activity through the lens of high-stakes testing, potentially limiting the motivational scope of digital resources. This is compounded by intense academic pressure, which can transform the autonomy of online learning into a source of anxiety rather than empowerment. Furthermore, profound regional and socioeconomic disparities in access mean that the modality itself (virtual or traditional) is not a neutral variable but one entangled with pre-existing inequality. Therefore, this study does not merely compare settings; it investigates how these contextual factors *interact* with learning modes to differentially influence psychological needs, engagement, and ultimately, outcomes for Chinese English learners.

Despite the growing body of literature on digital learning and language acquisition, significant research gaps remain. Previous studies have primarily focused on the effectiveness of online learning in Western contexts, with limited investigation into how these models function within the Chinese education system (Yue et al., 2025). Moreover, while prior research has explored the role of digital resources and teacher support in language learning, few studies have examined how engagement mediates the relationship between learning environments and learning outcomes in China (Han and Xu, 2025). Furthermore, existing studies often overlook the moderating effects of learner proficiency levels, technological accessibility, and instructional strategies, which may significantly influence learning motivation and self-regulation (Warschauer, 2004; Van Dijk, 2006; Chou et al., 2025). To bridge these gaps, this study aims to provide empirical insights into how diverse learning resources and support systems impact Chinese English language learners in both virtual and traditional settings.

This study contributes to the existing body of knowledge by integrating Self-Determination Theory (Ryan and Deci, 2000a) and Cognitive Load Theory (Sweller and Chandler, 1991) to explain how learning environments shape students’ motivation, engagement, and academic performance. Methodologically, it employs a quantitative research approach, utilizing Structural Equation Modeling (SEM) to establish causal relationships between variables. The findings of this study will provide valuable implications for educators, policymakers, and curriculum developers seeking to optimize English language learning strategies in China. The structure of this paper is as follows: Section 2 reviews relevant literature on digital learning resources, support systems, and learning motivation; Section 3 outlines the research methodology, including sample selection, data collection, and analytical techniques; Section 4 presents the findings and discussion; and Section 5 concludes with practical recommendations and future research directions.

### 1.1 Research problem statement and gap analysis

English language acquisition among Chinese learners has been a crucial focus in both academic and policy discussions, given the increasing demand for bilingual proficiency in globalized economies (Sadaf, 2025). However, disparities exist in the effectiveness of different learning environments—virtual versus traditional settings—in fostering motivation, self-regulation, and overall learning outcomes. While studies have examined online learning effectiveness (Sinaga, 2025) limited research has comparatively analyzed how diverse learning resources and support systems interact with learning modes to shape students’ engagement and performance (Faro et al., 2025). The gap remains in understanding how the interplay between instructional strategies, digital accessibility, and learning support influences English learning motivation and academic success, particularly in China’s evolving educational landscape.

Furthermore, a critical nuance within the Chinese digital learning landscape is the variety of settings where online English learning occurs. Existing research often treats ‘online learning’ as a monolithic category, overlooking significant distinctions between formal, institutionally-mandated e-learning platforms, commercial tutoring applications, and informal, self-directed learning via social media or content platforms like Bilibili and WeChat study groups. These environments differ radically in their structure, level of teacher interaction, peer community, and alignment with exam goals. The lack of specificity regarding where learning takes place online limits our understanding of how contextual factors, interact with resources and support systems to affect motivation and self-regulation. Therefore, a more granular analysis is required.

Existing literature highlights the challenges faced by Chinese English language learners (CELs) due to rigid educational structures and a test-oriented system, which often fail to incorporate diverse learning resources and support mechanisms that enhance intrinsic motivation and self-regulation (Gao and Zuo, 2025). Many studies have focused on the effectiveness of either virtual or traditional learning settings in isolation, neglecting a comparative perspective (Goroba et al., 2025). Moreover, limited research has explored the mediating role of engagement in learning, which is critical for sustaining motivation and improving learning results in both contexts (Xiong, 2025). This study addresses these gaps by conducting a comparative analysis of learning resources, support systems, and engagement across virtual and traditional settings, thereby offering empirical insights into optimizing English language instruction for CELs.

The primary sample for this study comprises Chinese English language learners, including university students enrolled in English courses, secondary school students engaged in hybrid learning, and adult learners participating in online English programs. China’s English learning population is one of the largest globally, with an estimated 400 million individuals actively studying English (Roctus, 2025). University students constitute a critical demographic due to the role of English proficiency in academic success and career prospects. Secondary school students represent an essential segment as they experience dual learning environments, balancing online and traditional modes of instruction (Yeşilyurt and Caner, 2025). Meanwhile, adult learners, including working professionals, are increasingly relying on virtual platforms for English acquisition, further emphasizing the need to assess the effectiveness of different learning settings (Çeken and Taşkın, 2025).

This population is particularly relevant as it provides a comprehensive understanding of how different age groups and educational backgrounds respond to diverse learning resources and support systems in varying learning modes. By incorporating multiple learner segments, the study enhances its generalizability and practical implications. Understanding the experiences of these learners can inform policymakers and educators in designing effective English language programs that cater to varied learning needs, thus addressing existing challenges in motivation, self-regulation, and engagement. Through empirical analysis, this study aims to contribute valuable insights into the optimization of English language education in China, bridging the gap between research and pedagogical practice.

Consequently, virtual-based learning is highly anticipated as a strategic tool for scaling quality English instruction nationally. Yet, this ambition collides with the on-the-ground reality that education in rural and mountainous areas is often fundamentally limited by poor internet access. This access divide necessitates a critical investigation not only into which mode is more effective, but for whom it is even possible, and how support systems must be designed differently to account for this infrastructural inequity.

## 2 Literature review

### 2.1 Theoretical foundation: self-determination theory (SDT)

The Self-Determination Theory (SDT), introduced by Deci and Ryan (1985); Ryan and Deci (2000b), serves as the primary theoretical framework for this study. SDT posits that human motivation is driven by three fundamental psychological needs: autonomy, competence, and relatedness. When these needs are met, learners exhibit higher self-regulation and intrinsic motivation, ultimately improving academic outcomes. This theory is particularly relevant to Chinese English Language Learners (CELs), whose learning experiences are shaped by traditional teacher-centered instruction and high-stakes examination pressure (Salsabila et al., 2025). SDT provides the *scientific psychological blueprint* for why and how student-centered learning works. It explains that for high-quality motivation, engagement, and wellbeing to flourish, three innate psychological needs must be satisfied. SCL is the pedagogical model designed to meet these needs.

SDT aligns with China’s National Medium- and Long-Term Education Reform and Development Plan (2010–2020), which emphasizes student-centered learning and skill-based education. The Chinese government has also promoted blended and technology-enhanced learning environments to improve language proficiency and global competitiveness (Likoko and Wu, 2025). Given these policy directions, this study explores how SDT-driven educational strategies, such as autonomy-supportive teaching and personalized digital learning resources, can enhance CELs’ motivation and self-regulation in English learning.

#### 2.1.1 SDT and its relevance to English language learning in China

In the context of English as a Foreign Language (EFL) education in China, SDT is particularly relevant as it provides a framework for understanding how learning autonomy, support systems, and learning modes influence student motivation and engagement (Liu et al., 2025). The traditional Chinese education system is known for its teacher-centered approach and exam-oriented learning culture, which often results in extrinsically motivated students (Huan and Sanmugam, 2025). However, recent educational reforms have emphasized student-centered learning, active participation, and the integration of digital technologies, aligning with SDT principles (Bhardwaj et al., 2025).

The Chinese government’s National Medium- and Long-Term Education Reform and Development Plan (2010–2020) and subsequent policy initiatives promote blended learning, digital resource integration, and internationalized curricula (Cretu and Grosseck, 2025). These reforms aim to enhance students’ motivation, self-regulation, and engagement in language learning, making SDT an ideal theoretical foundation for examining how diverse learning resources, support systems, and learning modes influence Chinese EFL learners’ academic outcomes. Socioeconomic factors create a motivation-hostile environment. They systematically attack the very conditions psychological need satisfaction required for motivation to thrive and be sustained. Research that ignores these factors risks blaming the student whom lack motivation rather than diagnosing the systemic barriers that crush even the most ardent initial motivation and how to observe the outcomes that might be moderated by these pervasive socioeconomic filters.

### 2.2 Motivation for learning: the key dependent variable

#### 2.2.1 The concept of motivation in second language learning

Motivation is a fundamental factor influencing the success of second language acquisition (SLA). Defined as the internal drive that compels learners to engage in language learning activities, motivation is categorized into two types: intrinsic motivation (learning for enjoyment or personal growth) and extrinsic motivation (learning due to external rewards, such as exams or career benefits) (Al-Hoorie, 2025). Research has shown that students with high intrinsic motivation demonstrate better engagement, self-regulation, and long-term retention of language skills (Mohebbi, 2025).

#### 2.2.2 Motivation among Chinese English language learners (CELs)

In China, the motivation to learn English is often driven by extrinsic factors, including university entrance exams, employment opportunities, and social expectations (He, 2025). However, studies suggest that promoting autonomy and self-directed learning can enhance intrinsic motivation and long-term engagement (Slamet et al., 2025). For example, a study by Dahri et al. (2025) found that students who had access to diverse digital learning resources and autonomy-supportive learning environments displayed higher motivation and better learning outcomes compared to those in traditional rote-learning settings. This study seeks to build on these findings by examining how learning resources, support systems, and learning modes shape motivation among CELs.

If students go home to an environment that depletes their psychological needs such as; stress, scarcity, and pressure, the classroom must become a deliberate, structured environment that replenishes them. It will not be perfect. The autonomy will be constrained, the competence hard-won, and the relatedness tested by competition. But the goal is relative improvement, not perfect fulfillment. Even a small, reliable dose of autonomy, a single clear experience of competence, and a genuine connection can sustain motivation far better than a system that ignores these needs entirely. The study exploring what specific, actionable features of learning resources and support systems can best deliver these need-satisfying experiences within the very real constraints of the Chinese educational context.

### 2.3 Self-regulation in learning

#### 2.3.1 Defining self-regulation in education

Self-regulation refers to students’ ability to control their learning behaviors, including goal-setting, time management, and self-monitoring (Ateş and Polat, 2025). Research has established that strong self-regulation skills correlate with higher academic performance and learning efficiency (Cheng et al., 2025).

#### 2.3.2 Self-regulation and EFL learning in China

For CELs, self-regulation is particularly crucial in digital and blended learning environments, where students must independently manage their learning progress (Yang C. C. Y. et al., 2025). Studies indicate that self-regulated learners engage more effectively in language learning, leading to better comprehension, retention, and proficiency (Shafiee Rad, 2025). This study examines how learning resources, support systems, and learning modes impact students’ self-regulation abilities, ultimately influencing their academic success.

### 2.4 Learning results: academic performance, test scores, and language proficiency

#### 2.4.1 Measuring learning outcomes in EFL education

Learning results in EFL education are commonly measured by academic performance (course grades), test scores (standardized assessments), and language proficiency levels (Derakhshan and Fathi, 2025). Several studies have confirmed a strong relationship between motivation, self-regulation, and language learning outcomes (Firdaus et al., 2025).

#### 2.4.2 Factors affecting learning results in Chinese EFL context

A study by Pratiwi et al. (2025) found that students in blended learning environments achieved higher test scores compared to those in traditional classroom settings. Similarly, Mohebbi (2025) reported that learners with access to digital learning tools demonstrated greater improvement in language proficiency over time. This study seeks to explore whether learning mode, support systems, and resource availability contribute to enhanced learning results among CELs.

### 2.5 Independent variables and their influence on motivation and self-regulation

#### 2.5.1 Diverse learning resources and autonomy in learning

Diverse learning resources, including digital textbooks, multimedia content, AI-based language tutors, and interactive online exercises, provide learners with greater autonomy in their learning process (Hong and Guo, 2025). According to SDT, autonomy is a key driver of intrinsic motivation, as students who perceive control over their learning experience are more likely to engage deeply and persist in their studies (Mayangsari et al., 2025).

Recent studies have highlighted the positive impact of digital learning resources on language learning motivation. For instance, Yaseen et al. (2025) examined the effects of AI-powered personalized learning platforms on EFL learners in China, finding that students who used adaptive learning technologies demonstrated higher engagement and self-regulation skills than those in traditional classrooms. Similarly, Pramawati et al. (2025) found that multimedia-based learning environments improved students’ ability to retain vocabulary and comprehend complex texts. Given these findings, this study hypothesizes that diverse learning resources positively influence motivation and self-regulation among CELs.

#### 2.5.2 Support systems: teacher, peer, and parental support

Support systems are crucial in fostering competence and relatedness, two key SDT components that influence learning motivation (Molina et al., 2025). Teacher guidance, peer collaboration, and parental involvement serve as external motivators that reinforce students’ confidence and engagement in language learning (Khoso and Fattah Soomro, 2025).

Research has consistently shown that teacher autonomy-supportive behaviors—such as providing meaningful feedback and encouraging independent thinking—enhance students’ intrinsic motivation and learning persistence (Zhang and He, 2025). Similarly, Lestari et al. (2025) found that peer-assisted learning strategies increased students’ willingness to practice speaking and writing in English. Additionally, parental involvement, particularly in online learning environments, has been linked to higher levels of engagement and academic success among EFL learners (Andrada and Barrot, 2025).

This study explores how various support systems interact to enhance motivation and self-regulation in Chinese EFL learners and whether certain types of support are more effective in different learning environments.

#### 2.5.3 Learning mode: virtual vs. traditional settings

The learning mode (virtual, face-to-face, or hybrid) influences students’ motivation, engagement, and academic performance. Virtual learning environments provide flexibility and accessibility, allowing students to tailor their learning experiences to their needs (Berezi, 2025). However, they also require strong self-regulation skills, as the lack of direct teacher supervision can lead to decreased motivation (Sarwar et al., 2025).

Studies comparing virtual and traditional EFL learning have yielded mixed results. For example, Bensalem et al. (2025) found that students in blended learning environments achieved better language proficiency outcomes than those in fully online or fully traditional settings. Another study by Sherov (2025) reported that students who participated in interactive virtual classrooms showed higher engagement and motivation compared to those in passive lecture-based online courses. Given these findings, this study investigates the impact of different learning modes on CELs’ motivation and self-regulation. In this study, the term ‘virtual learning setting’ refers specifically to formal, institutionally-facilitated online learning conducted via a designated Learning Management System (LMS). ‘Moodle,’ ‘Yu Class’, and Chaoxing Learning Tong’.

### 2.6 Mediating role of learning engagement

Learning engagement, defined as active participation, cognitive involvement, and emotional commitment to learning, mediates the relationship between the independent variables (diverse learning resources, support systems, learning mode) and the dependent variables (motivation, self-regulation, learning outcomes) (Sherov, 2025). Higher engagement levels correlate with improved academic performance and sustained motivation (Chen, 2025).

Research has shown that students who actively participate in learning activities, whether through collaborative projects, online discussions, or interactive simulations, develop stronger self-regulation skills and motivation (Yorganci, 2025). This study examines whether engagement acts as a mediating factor that enhances the positive effects of learning resources, support systems, and learning modes on motivation and self-regulation.

### 2.7 Hypotheses development

In line with structural equation modeling conventions, this study distinguishes between direct-effect hypotheses and indirect (mediation) hypotheses. Direct hypotheses specify the immediate relationships between independent variables and outcome variables, while mediation hypotheses examine whether these relationships are transmitted through engagement in learning. Explicitly stating both types of hypotheses ensures conceptual clarity and alignment between the theoretical framework and the empirical structural model (Hair and Alamer, 2022). Based on the literature review, the following hypotheses are proposed:

Direct Effect Hypotheses (Antecedents of Engagement)

*H1:* Diverse Learning Resources (DLR) have a significant effect on Engagement in Learning (EL).

*H2:* Support Systems (SS) have a significant effect on Engagement in Learning (EL).

*H3:* Learning Mode (LM) has a significant effect on Engagement in Learning (EL).

*H4:* Engagement in Learning (EL) has a significant effect on Motivation for Learning (ML).

*H5:* Engagement in Learning (EL) has a significant effect on Self-Regulation (SR).

*H6:* Engagement in Learning (EL) has a significant effect on Learning Results (LR).

Indirect (Mediating) Effect Hypotheses

*H7:* Engagement in Learning (EL) mediates the relationship between Diverse Learning Resources (DLR) and Motivation for Learning (ML).

*H8:* Engagement in Learning (EL) mediates the relationship between Support Systems (SS) and Motivation for Learning (ML).

*H9:* Engagement in Learning (EL) mediates the relationship between Learning Mode (LM) and Motivation for Learning (ML).

*H10:* Engagement in Learning (EL) mediates the relationship between Diverse Learning Resources (DLR) and Self-Regulation (SR).

*H11:* Engagement in Learning (EL) mediates the relationship between Support Systems (SS) and Self-Regulation (SR).

*H12:* Engagement in Learning (EL) mediates the relationship between Learning Mode (LM) and Self-Regulation (SR).

*H13:* Engagement in Learning (EL) mediates the relationship between Diverse Learning Resources (DLR) and Learning Results (LR).

*H14:* Engagement in Learning (EL) mediates the relationship between Support Systems (SS) and Learning Results (LR).

*H15:* Engagement in Learning (EL) mediates the relationship between Learning Mode (LM) and Learning Results (LR).

The inclusion of direct paths from the independent variables to both Engagement in Learning and the outcome variables follows recommended mediation testing procedures in SEM, allowing the assessment of whether indirect effects via engagement operate as complementary or competitive mediation. Accordingly, the direct effects reported in Table 1 should be interpreted as part of the mediation assessment rather than as isolated relationships.

**Table 1:** Hypotheses for the direct effect.

Hypotheses	Original sample (O)	Sample mean (M)	Standard deviation (STDEV)	*T* statistics (|O/STDEV|)	*p* values
H1: DLR → EL	−0.546	−0.541	0.075	7.303	0
H2: SS → EL	0.43	0.434	0.073	5.903	0
H3: LM → EL	−0.012	−0.01	0.049	0.243	0.808
H4: EL → ML	0.313	0.305	0.061	5.113	0
H5: EL → SR	0.401	0.394	0.074	5.408	0
H6: EL → LR	0.005	0.004	0.033	0.137	0.891

The green colour means the *p* value is significant at *p* < 0.05. The red colour is not significant.

To ensure transparency and alignment between the conceptual framework and the empirical model, all structural paths tested in the SEM were explicitly specified as part of the hypothesized model. In particular, the direct paths from Diverse Learning Resources (DLR), Support Systems (SS), and Learning Mode (LM) to Engagement in Learning (EL) and the outcome variables (ML, SR, LR) were treated as theory-driven hypotheses rather than *post hoc* or exploratory additions, even when not each path was individually labeled with a separate hypothesis number. Accordingly, the reported direct and indirect effects should be interpreted as confirmatory tests of the integrated SDT- and engagement-based model rather than as unplanned exploratory analyses.

## 3 Methodology

### 3.1 Research design

This study employs a quantitative research design to examine the relationships between diverse learning resources, support systems, and learning modes on motivation for learning, self-regulation, and learning outcomes among Chinese English Language Learners (CELs). A cross-sectional survey approach was selected, allowing for the collection of data from a large sample within a specific timeframe while ensuring statistical robustness and generalizability. The Structural Equation Modeling (SEM) technique was applied to test the hypothesized relationships, as it is particularly well-suited for assessing complex causal models in educational research (Hair and Alamer, 2022). Given that SEM provides more precise estimations of both direct and indirect effects among study variables, it was deemed appropriate for this investigation, allowing for the validation of both measurement and structural models (Sadenova et al., 2025).

### 3.2 Population and sampling

The study’s target population consists of Chinese English Language Learners (CELs) across various educational settings. This population was selected based on its relevance to second-language acquisition research, particularly in understanding the interplay between learning resources, support systems, and motivation in English proficiency development. The sample includes university students enrolled in English for Academic Purposes (EAP), Business English, and General English courses, secondary school students actively engaged in dual-learning environments that combine online and face-to-face instruction, and adult learners participating in online English language programs for career advancement. The Overall higher education eligible learners were 10,000. The male contributed 40% while the female contributed 60% of the sample size. The questionnaire collected using *Wenjuanxing* platform.

A total of 400 survey questionnaires were distributed, ensuring an appropriate sample size for SEM analysis, as determined by Cohen (2016) power analysis. Out of these, 300 completed responses were obtained, yielding a response rate of 75%, which is considered acceptable for survey-based educational research (Sataloff and Vontela, 2021). A proportionate stratified random sampling technique was employed to ensure adequate representation across university students, secondary school learners, and adult learners, thereby mitigating sampling bias and enhancing the external validity of the findings (McEwan, 2020).

### 3.3 Data collection procedure

Data collection was conducted over a three-month period, from January to March 2024, utilizing a structured self-administered online survey. The survey was distributed via university portals, online English language learning platforms, and professional education networks in China. The online survey method was chosen due to its accessibility and efficiency in reaching a diverse population, particularly adult learners and university students who frequently engage in digital learning environments (Kumi-Yeboah et al., 2020).

To ensure the clarity and reliability of the questionnaire, a pilot study involving 30 respondents was conducted prior to the full-scale survey distribution. The pilot test assessed the comprehensibility, internal consistency, and reliability of the instrument, and minor modifications were made based on participant feedback to improve readability and measurement precision. The revised survey was then finalized and deployed, ensuring its suitability for large-scale data collection.

### 3.4 Measures and instruments

The study’s measurement instrument consisted of validated scales adapted from prior research on motivation, self-regulation, and learning outcomes in second-language education. All items were measured using a five-point Likert scale ranging from 1 (strongly disagree) to 5 (strongly agree). This scaling method was selected due to its effectiveness in capturing participant perceptions regarding the quality of learning resources, support systems, learning modes, and their impacts on motivation, self-regulation, and academic performance.

The independent variable Diverse Learning Resources was measured using a modified version of the Technology-Enhanced Learning Scale (TELS), as proposed by Pickering and Swinnerton (2019) which assesses the effectiveness of digital textbooks, multimedia content, and interactive exercises in English language learning. Support Systems were measured using an adapted Teacher and Peer Support Scale (TPSS), which evaluates the role of teacher guidance, peer support, and parental involvement in fostering learning motivation (Olana and Tefera, 2022). Learning Mode, which distinguishes between virtual and traditional learning settings, was assessed using a binary categorical measure, supplemented by additional items examining perceived effectiveness in both learning environments (Khoudi et al., 2025).

Dependent variables were measured using well-established scales. Motivation for Learning was assessed using the Intrinsic and Extrinsic Motivation Scale (IEMS) developed by Wu et al. (2025) which evaluates the extent to which students are driven by internal satisfaction versus external rewards in English learning. Self-Regulation was measured using the Self-Regulated Learning Strategies Scale (SRLSS), which captures goal-setting, time management, and self-monitoring behaviors essential for academic success (Tanimura et al., 2023). Learning Outcomes were evaluated based on self-reported academic performance, English test scores, and language proficiency levels measured using the Common European Framework of Reference for Languages (CEFR) (Vonkova et al., 2023).

**Figure 1:**
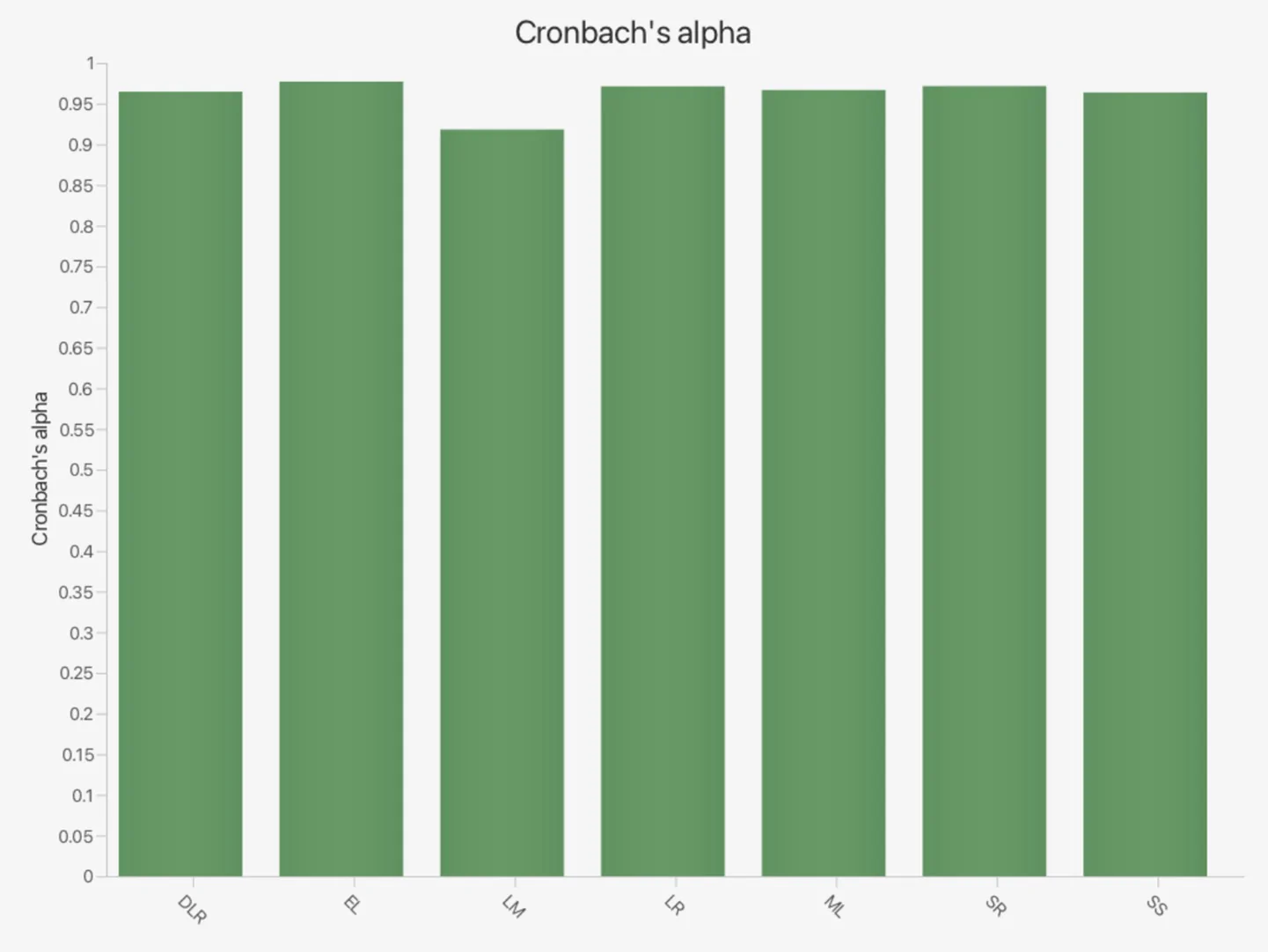
Cronbach’s alpha.

**Figure 2:**
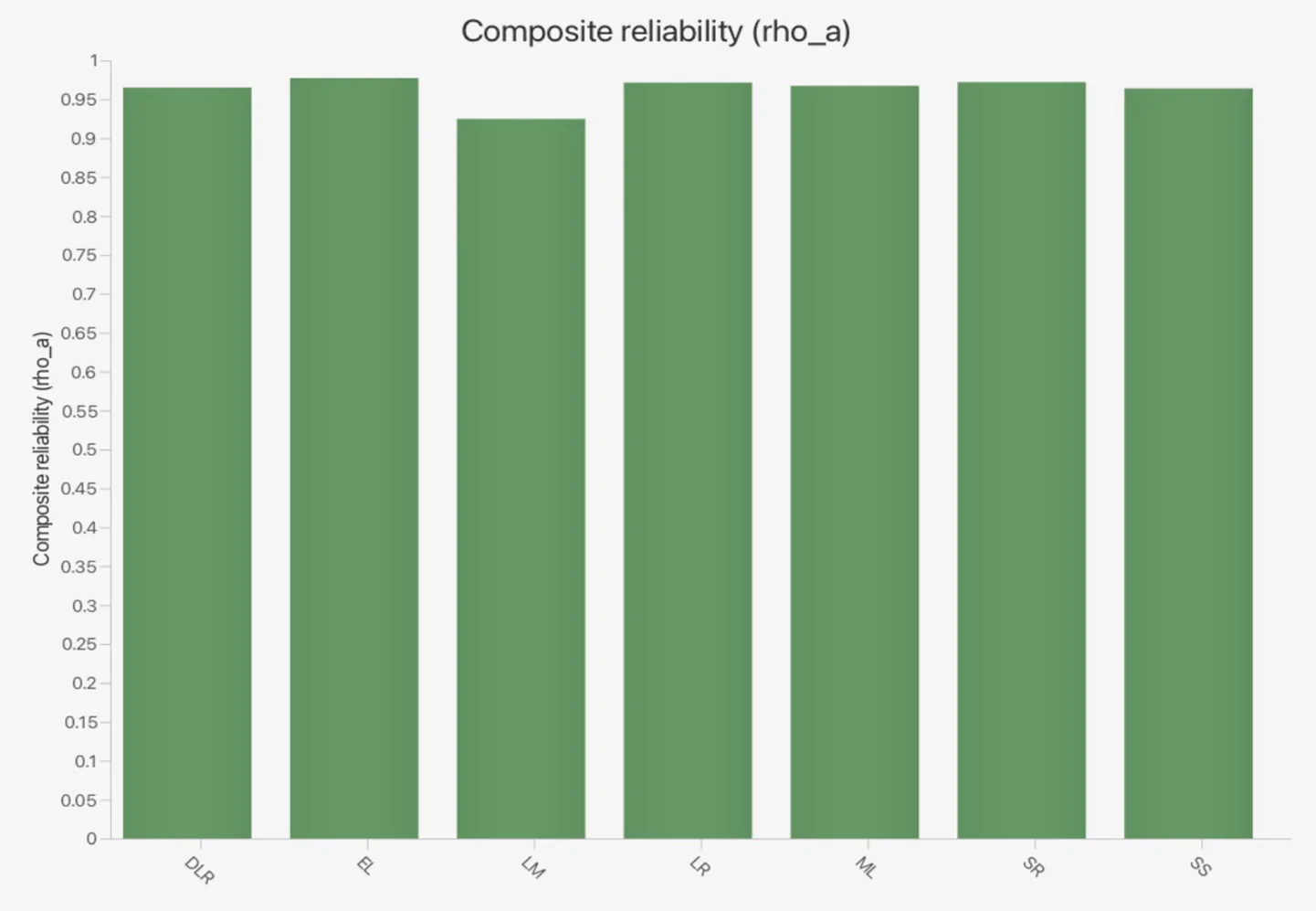
Composite reliability (rho_a).

**Figure 3:**
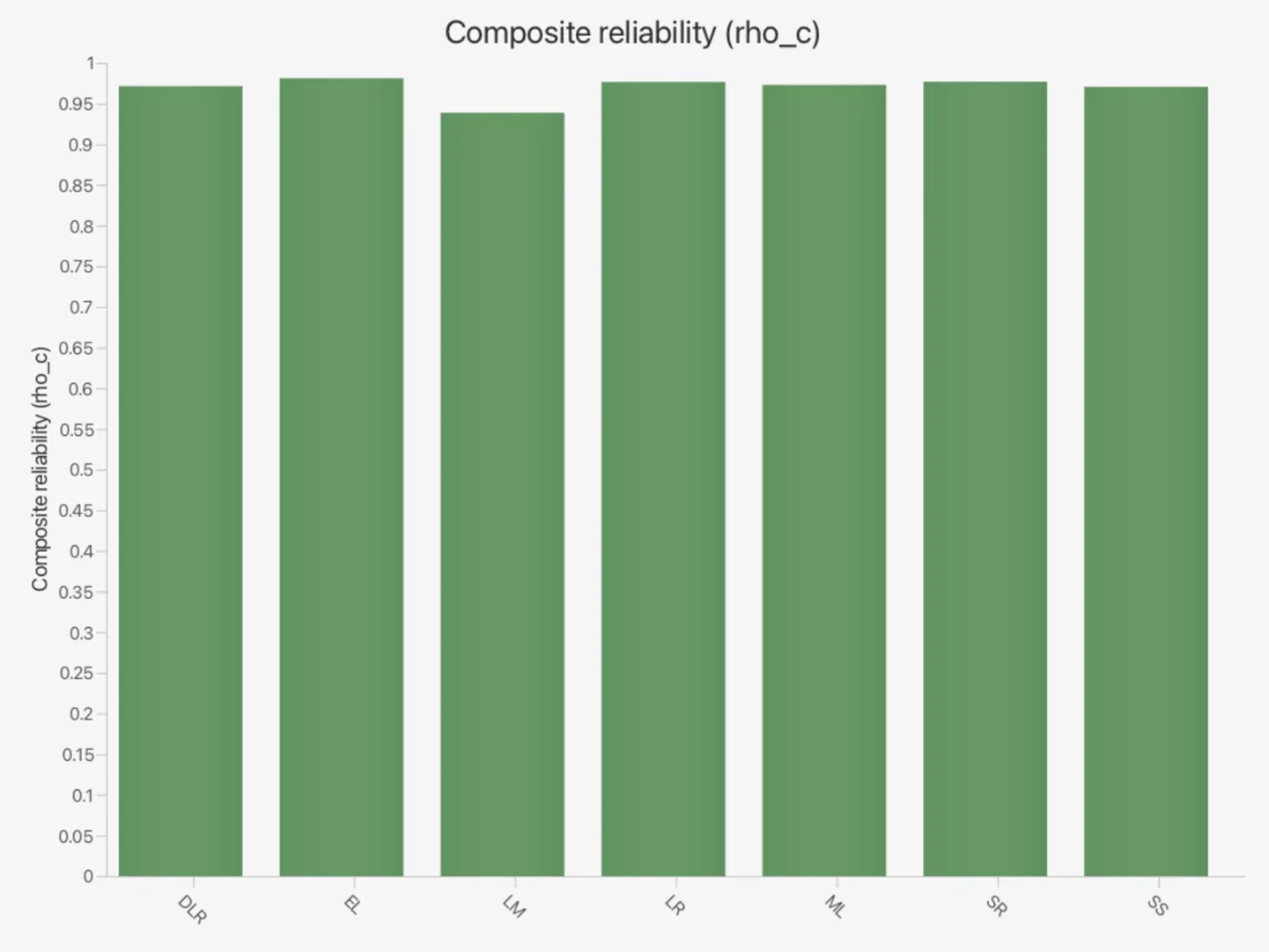
Composite reliability (rho_c).

**Figure 4:**
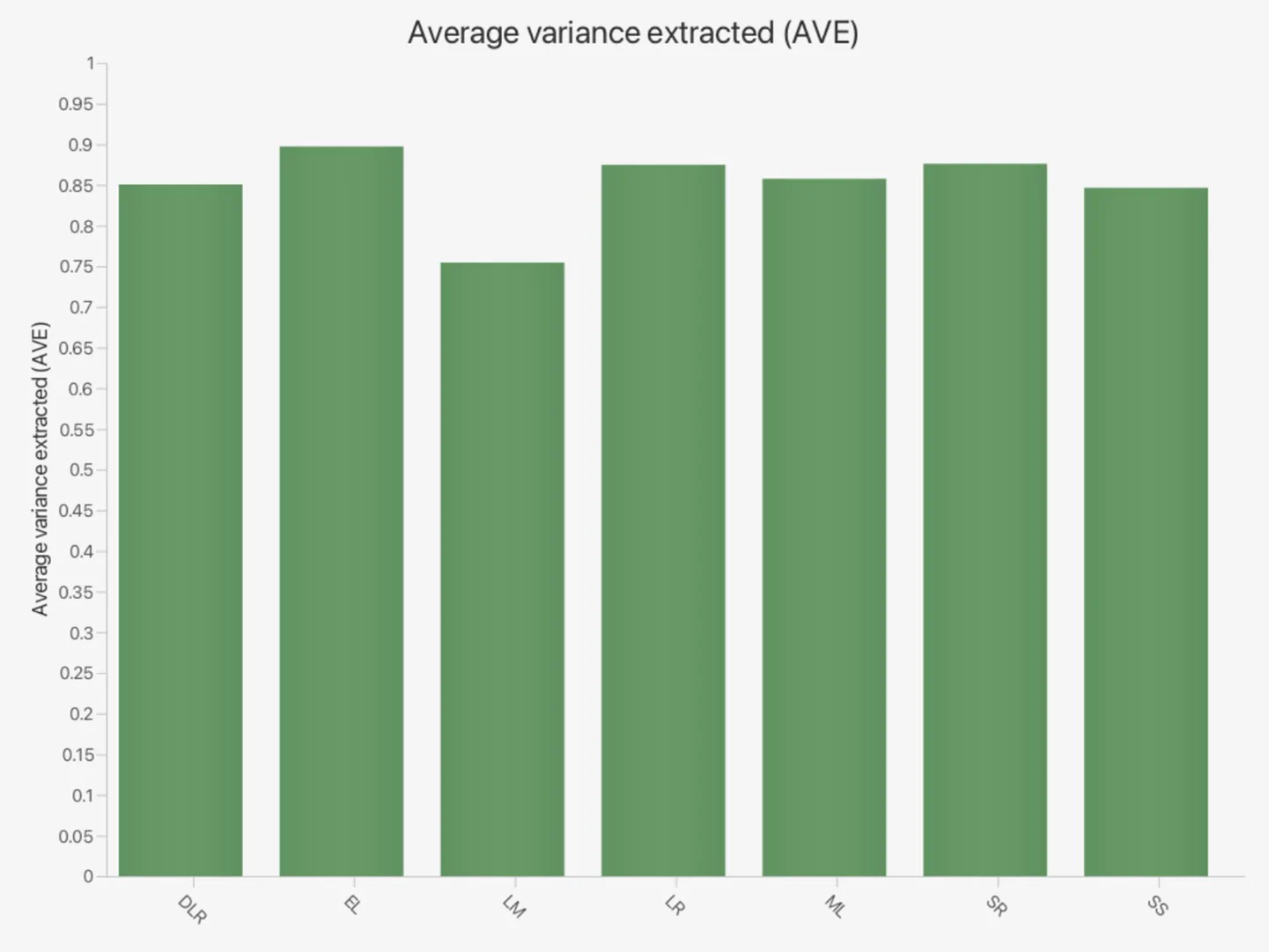
Average variance extracted (AVE).

The mediating variable, Learning Engagement, was measured using the Student Engagement in Learning Scale (SELS), developed by Kim et al. (2024) which assesses active participation, cognitive involvement, and interaction levels in both online and face-to-face learning environments. All scales demonstrated strong reliability (Cronbach’s *α* > 0.80) and construct validity, ensuring the robustness of the measurement model. Confirmatory Factor Analysis (CFA) was conducted to validate the factor structure prior to hypothesis testing (Figures 1–4).

### 3.5 Data analysis

Prior to hypothesis testing, data screening and preliminary analyses were conducted to identify missing values, outliers, and normality violations. Mahalanobis distance analysis was applied to detect outliers, while Little’s MCAR test confirmed that missing data were missing completely at random (Leys et al., 2018).

The main statistical analyses were conducted using Structural Equation Modeling (SEM) with SmartPLS 4.0, following a two-step approach. First, the measurement model assessment examined construct reliability, convergent validity (ensuring an Average Variance Extracted [AVE] greater than 0.50), and discriminant validity using the Fornell-Larcker criterion (Roemer et al., 2021). Second, the structural model assessment evaluated path coefficients, *R*^2^ values, and effect sizes (*f*^2^) to determine the significance and magnitude of hypothesized relationships (Buafra and Salahudin, 2022). The model’s goodness-of-fit was assessed using SRMR (<0.08), RMSEA (<0.06), and CFI (>0.90), ensuring compliance with SEM fit criteria (Rodríguez-Díaz et al., 2025).

### 3.6 Ethical considerations

Ethical approval for this study was obtained from the relevant Institutional Review Board (IRB), ensuring adherence to ethical guidelines for human research (American Psychological Association, 2023). All participants provided informed consent, which included details on voluntary participation, data confidentiality, and the right to withdraw at any stage without consequences. Given that the study involved an online survey, stringent data security measures were implemented to protect participant anonymity and prevent unauthorized access. Responses were anonymized before analysis, and access to the dataset was restricted solely to the research team. Additionally, the study adhered strictly to ethical principles of non-coercion, minimal risk, and fair treatment, ensuring full compliance with established ethical research standards (Gopalan et al., 2025). Ethical approval for this study was obtained from the relevant Institutional Review Board (IRB Approval No. IRB-2024-00123), ensuring full compliance with established ethical guidelines for research involving human participants.

## 4 Results and discussion

**Figure 5:**
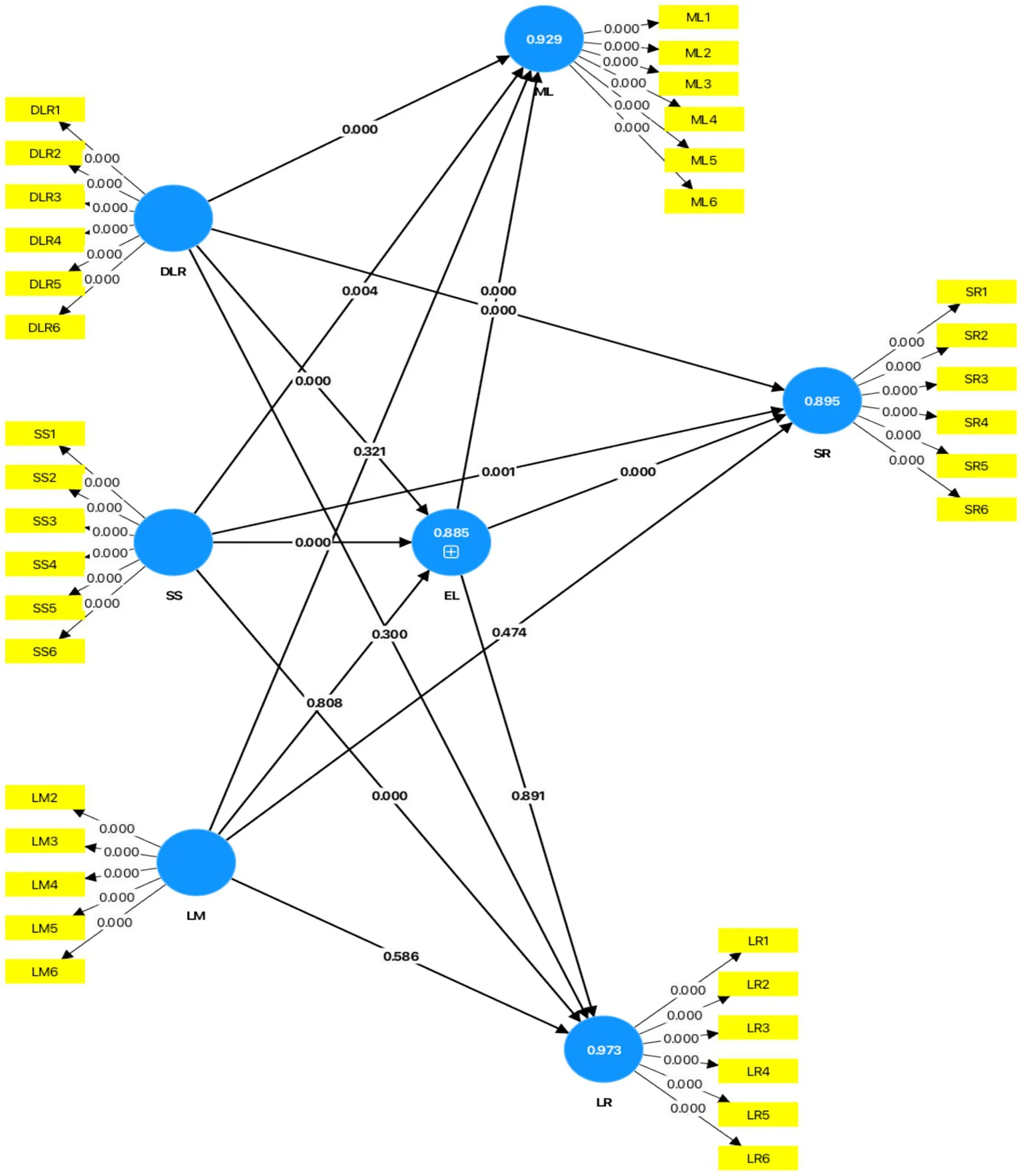
Graphical output of structural equation model of this study—OL, *p*-value, *R*^2^ adjusted.

The empirical analysis tests the complete hypothesized structural model presented in Figure 5 (H1–H15), which links learning environment factors (DLR, SS, LM) to outcomes (ML, SR, LR) through engagement mediation. Consistent with the research questions and theoretical framework outlined in Sections 1 and 2, Structural Equation Modeling via SmartPLS 4.0 evaluates both direct and indirect effects, providing a rigorous assessment of the proposed pathways.

### 4.1 Descriptive statistics and demographic analysis

The study collected data from a total of 400 Chinese English Language Learners across different educational backgrounds, with 300 valid responses retained for analysis. The sample included university students enrolled in English courses such as English for Academic Purposes, Business English, and General English, as well as secondary school students actively learning English in dual learning environments and adult learners participating in online English programs for career advancement. Descriptive analysis indicated a diverse representation of learners across various age groups, learning settings, and language proficiency levels.

Table 2 presents the demographic characteristics of the respondents included in the final analysis. The sample comprised a higher proportion of female learners (60%) compared to male learners (40%). Participants were drawn from diverse educational backgrounds, including secondary school students, university students, and adult learners, ensuring broad representation across different learning stages. The respondents exhibited varying levels of English proficiency, with the majority classified as intermediate learners. In terms of learning mode, both virtual and traditional learning settings were well represented. Additionally, participants were recruited from urban and semi-urban regions across China, supporting the geographic diversity and representativeness of the sample (Tables 3–7).

**Table 2:** Demographic characteristics of respondents (*N* = 300).

Variable	Category	Frequency (*n*)	Percentage (%)
Gender	Male	120	40.0
	Female	180	60.0
Educational level	Secondary school students	90	30.0
	University students	150	50.0
	Adult learners	60	20.0
English proficiency level	Beginner	72	24.0
	Intermediate	150	50.0
	Advanced	78	26.0
Learning mode	Virtual learning	165	55.0
	Traditional learning	135	45.0
Geographic location	Urban regions	186	62.0
	Semi-urban regions	114	38.0

Percentages may not total 100% due to rounding.

**Table 3:** Reliability and convergent validity assessment of measurement constructs.

Construct	Cronbach’s alpha	Composite reliability (rho_a)	Composite reliability (rho_c)	AVE
DLR	0.965	0.965	0.972	0.851
EL	0.977	0.977	0.981	0.897
LM	0.918	0.925	0.939	0.755
LR	0.971	0.971	0.977	0.875
ML	0.967	0.967	0.973	0.858
SR	0.972	0.972	0.977	0.876
SS	0.964	0.964	0.971	0.847

**Table 4:** Discriminant validity assessment using inter-construct correlations.

Constructs	AALS	DIA	ILT	LEM	LOCLP	LSCD
AALS	–					
DIA	0.891	–				
ILT	0.886	0.845	–			
LEM	0.876	0.784	0.765	–		
LOCLP	0.848	0.773	0.766	0.755	–	
LSCD	0.849	0.785	0.764	0.763	0.751	–

**Table 5:** Coefficient of determination (*R*^2^ and Adjusted *R*^2^) for endogenous constructs.

Dependent variable	*R*-square	Adjusted *R*-square
EL (Engagement in Learning)	0.885	0.883
LR (Learning Results)	0.973	0.972
ML (Motivation for Learning)	0.929	0.927
SR (Self-Regulation)	0.895	0.892

**Table 6:** Assessment of effect sizes (*f*^2^) for structural model pathways.

Pathway	*f*^2^ value	Effect size interpretation
DLR → EL	0.525	Large
EL → LR	4.261	Large
EL → ML	7.126	Large
EL → SR	6.068	Large
LM → EL	0.000	No effect
SS → EL	0.249	Medium

**Table 7:** Indirect relationship—mediation.

Total effect			Direct effect			Indirect effect							Hypothesis result
Coefficient	*T* value	*p* value	Coefficient	*T* value	*p* value	Hypothesis	Coefficient	SE	*T* value	*p* value	Percentile Bootstrap 95% CI		
											Lower	Upper	
−0.171	3.866	0.000	−0.453	5.624	0.000	H7: DLR → EL → ML	−0.512	0.069	7.452	0.000	−0.626	−0.355	Competitive partial mediation
0.135	4.399	0.000	0.263	2.883	0.004	H8: SS → EL → ML	0.413	0.068	6.066	0.000	0.282	0.546	Complementary partial mediation
−0.004	0.243	0.808	−0.044	0.993	0.321	H9: LM → EL → ML	−0.025	0.045	0.554	0.580	−0.126	0.055	No mediation
−0.219	4.098	0.000	−0.349	4.397	0.000	H10: DLR → EL → SR	0.169	0.042	3.995	0.000	−0.622	−0.350	Complementary partial mediation
0.173	4.176	0.000	0.249	2.883	0.004	H11: SS → EL → SR	−0.256	0.054	4.738	0.000	0.279	0.543	Competitive partial mediation
−0.005	0.240	0.811	−0.029	0.716	0.474	H12: LM → EL → SR	0.006	0.021	0.272	0.785	−0.125	0.054	No mediation
−0.002	0.137	0.891	−0.031	1.037	0.300	H13: DLR → EL → LR	−0.492	0.060	8.159	0.000	−0.588	−0.349	Full mediation
0.002	34.872	0.000	0.962	34.872	0.000	H14: SS → EL → LR	0.397	0.070	5.644	0.000	0.265	0.538	Complementary partial mediation
−0.000	0.545	0.586	−0.008	0.545	0.586	H15: LM → EL → LR	−0.024	0.043	0.555	0.579	−0.120	0.053	No mediation

The green colour means the *p* value is significant at *p* < 0.05. The red colour is not significant.

### 4.2 Construct reliability and validity

To ensure the robustness of the measurement model, construct reliability and validity were assessed using Cronbach’s alpha, composite reliability (rho_a and rho_c), and average variance extracted (AVE). The results indicate strong internal consistency across all constructs, with Cronbach’s alpha values ranging from 0.918 to 0.977, exceeding the recommended threshold of 0.7 (Hair and Alamer, 2022). Composite reliability values (rho_c) were all above 0.9, indicating high construct reliability. The AVE values ranged from 0.755 to 0.897, surpassing the 0.50 benchmark, confirming convergent validity (Fornell and Larcker, 1981).

These results confirm that all constructs exhibit high reliability and validity, ensuring the measurement model is well-specified for further analysis.

### 4.3 Discriminant validity assessment and HTMT ratio

To assess discriminant validity, the Heterotrait-Monotrait (HTMT) ratio of correlations was examined. The common threshold for HTMT is <0.90, with a more conservative threshold of <0.85 applied in cases where constructs are conceptually similar (Henseler et al., 2015). The HTMT values ranged from 0.751 to 0.891, demonstrating that all constructs maintained discriminant validity.

Since all HTMT values fall below the 0.90 threshold, discriminant validity is established, confirming that each construct is empirically distinct from the others.

### 4.4 Model fit and structural equation modeling (SEM) readiness

To ensure the suitability of the dataset for structural equation modeling using SEM-PLS, the *R*-square and adjusted *R*-square values were examined. The results indicate a strong explanatory power of the independent variables on the dependent variables.

These values demonstrate a strong model fit, confirming the research framework’s predictive capability.

### 4.5 Effect size analysis (*f*^2^)

The f-square values indicate the impact of predictor variables on endogenous variables based on Cohen (1988) effect size interpretation. The analysis revealed significant relationships among key variables.

The findings indicate that Engagement in Learning (EL) significantly impacts Learning Results (LR), Motivation for Learning (ML), and Self-Regulation (SR). The path from Diverse Learning Resources (DLR) to EL also demonstrates a substantial effect. However, Learning Mode (LM) does not significantly influence Engagement in Learning, suggesting that other contextual factors may play a stronger role in shaping engagement levels.

The results confirm that the research model exhibits strong measurement properties, ensuring validity and reliability for further structural analysis. The discriminant validity assessment demonstrates that all constructs are distinct, while the high *R*-square values indicate a well-fitting model. Effect size analysis further highlights the significant relationships between key constructs, emphasizing the importance of Engagement in Learning as a mediating variable. These findings provide a solid foundation for conducting SEM-PLS to test the hypothesized relationships and advance understanding of motivation, self-regulation, and learning outcomes among Chinese English Language Learners.

The bar charts collectively present the reliability, and validity, of the structural equation model employed in this study. The path coefficient chart visually represents the magnitude and direction of hypothesized relationships, confirming which independent variables significantly influence the dependent variable. Strong positive or negative coefficients indicate robust relationships, while weaker values suggest limited impact. Furthermore, the reliability indicators, including Cronbach’s alpha and composite reliability (rho_a and rho_c), ensure that the constructs exhibit strong internal consistency, affirming the dependability of the measurement model. The average variance extracted (AVE) values validate the discriminant and convergent validity of the constructs, confirming that the latent variables adequately capture the intended theoretical constructs. Together, these statistical measures reinforce the robustness and suitability of the model for hypothesis testing and theoretical validation. The results highlight the empirical strength of the relationships within the conceptual framework and provide a foundation for deeper theoretical discussion.

The suggested beneficial relationship between various learning resources and engagement was not confirmed; however, the significant negative path coefficient (*β* = −0.453) presents a considerable opportunity for theoretical advancement and methodological assessment. Rather than dismissing the data as anomalous, it necessitates a deeper analysis that could clarify the complex dynamics of learning environments. This may occur due to several factors, including multicollinearity with other model components or measurement overlap, when the definition of diversity inadvertently include information overload or cognitive disorientation. This unexpected result may suggest a genuine, non-linear relationship where an excess of options or resource fragmentation impedes focused involvement, a phenomenon acknowledged in the literature on the paradox of choice. A thorough analysis of these choices is essential to move beyond a superficial conflict, transforming an unexpected result into a meaningful contribution that enhances both the readers’ understanding and the intellectual depth of the field.

### 4.6 Structural equation model

This comprehensive set of hypotheses (H1–H15) directly addresses the study’s primary research questions by specifying (1) the direct effects of diverse learning resources, support systems, and learning mode on student engagement (H1–H3); (2) the effects of engagement on key psychological and performance outcomes (H4–H6); and (3) the mediating role of engagement in transmitting structural learning environment factors to motivation, self-regulation, and learning results (H7–H15). Figure 5 visually represents this complete hypothesized structural model, which integrates Self-Determination Theory with an engagement mediation framework to test how educational inputs drive motivational and performance outcomes among Chinese English language learners. The subsequent empirical analysis via Structural Equation Modeling rigorously evaluates all specified paths, ensuring alignment between theoretical propositions, model specification, results, and their interpretation in the discussion.

The results confirm key theoretical propositions from Self-Determination Theory while revealing nuanced mediation patterns in the tested model (Figure 5, H1–H15). Specifically, support systems enhance engagement and outcomes through complementary partial mediation (H2, H8, H11, H14), whereas diverse learning resources exhibit competitive partial mediation due to their unexpected negative direct effect on engagement (H1, H7, H10, H13). These findings establish a coherent narrative linking structural learning factors to psychological needs satisfaction, engagement, and performance outcomes among Chinese English language learners.

In relation to the stated hypotheses, the results provide a clear pattern. H1 and H2 are supported, as both Diverse Learning Resources (DLR) and Support Systems (SS) show significant direct effects on Engagement in Learning (EL), although the direction of the DLR → EL path is unexpectedly negative. H3 is not supported, because Learning Mode (LM) does not exhibit a significant direct effect on EL. H4, H5, and H6 are supported, given that EL has significant positive effects on Motivation for Learning (ML), Self-Regulation (SR), and Learning Results (LR). Regarding the mediating hypotheses, H7–H8 and H10–H11 are supported, indicating that EL partially mediates the effects of DLR and SS on ML and SR, while H9 and H12 are not supported due to the nonsignificant role of LM. Finally, the results provide partial support for H13–H15, with EL mediating the relationships between DLR and SS and Learning Results (LR), whereas mediation via LM is not significant. These hypothesis-specific findings form the basis for the thematic discussion of competitive and complementary partial mediation reported below.

The direct effect results in Table 1 reveal important insights about the structural relationships among the study variables and serve as a foundation for interpreting the mediation model. First, the independent variables Diverse Learning Resources (DLR) and Support Systems (SS) demonstrate significant direct effects on Engagement in Learning (EL), whereas Learning Mode (LM) does not exhibit a significant effect. Specifically, DLR shows a strong negative effect on EL (*β* = −0.546, *p* < 0.001), suggesting that the mere presence of diverse resources may overwhelm learners unless accompanied by effective integration and guidance. This finding is consistent with recent empirical work indicating that excessive resource diversity can increase cognitive load and reduce engagement when learners lack clear strategies for navigation and use (Skulmowski and Xu, 2022). Conversely, SS exerts a significant positive effect on EL (*β* = 0.430, *p* < 0.001), confirming that institutional support mechanisms such as instructor guidance, feedback, and peer support are critical for fostering active engagement in learning activities, aligning with contemporary research that underscores the centrality of structured support in enhancing engagement and persistence in educational contexts (Guo et al., 2025).

Furthermore, the pattern of direct effects from Engagement in Learning to the key learning outcomes provides nuanced evidence about the role of engagement as a proximal determinant of motivational, self-regulatory, and performance outcomes. The results show that EL has significant positive direct effects on Motivation for Learning (ML) (*β* = 0.313, *p* < 0.001) and Self-Regulation (SR) (*β* = 0.401, *p* < 0.001), indicating that higher levels of engagement are associated with stronger intrinsic motivation and more effective self-regulatory behaviors among learners. These findings echo recent theoretical assertions that engagement functions as a dynamic catalyst for both motivational orientations and self-regulatory processes, reinforcing students’ investment in learning tasks and their ability to manage cognitive and affective dimensions of learning (Alam and Mohanty, 2024). The significant path coefficients for EL → ML and EL → SR align with the broader literature emphasizing the mediating role of engagement in translating environmental and pedagogical inputs into positive learner dispositions and regulatory competencies (Gao et al., 2024).

In contrast, the direct effect of EL on Learning Results (LR) was not statistically significant (*β* = 0.005, *p* = 0.891), suggesting that engagement in isolation may not directly translate into measurable learning outcomes such as test performance or objective learning gains. This non-significant finding implies that while engagement influences motivational and self-regulatory processes, its impact on observable learning results might be contingent on additional factors such as assessment design, instructional quality, or the alignment between instructional activities and evaluation criteria. Recent studies have similarly observed that engagement alone does not guarantee superior performance outcomes unless it is coupled with effective instructional mediation and contextual supports that facilitate the transfer of engagement into performance gains (Liu et al., 2023). Taken together, the direct effect results in Table 1 underscore the complexity of the engagement–outcome relationship, highlighting the importance of distinguishing between proximal psychological processes and distal performance measures in educational research.

The structural model assessment in Figure 5 demonstrates the relationships between diverse learning resources (DLR), support systems (SS), and learning modes (LM) in predicting motivation for learning (ML), with engagement (E) serving as a mediator. The path coefficients and significance levels indicate that diverse learning resources negatively influence engagement (*β* = −0.453, *t* = 5.624, *p* = 0.000), while support systems exhibit a positive and significant impact on engagement (*β* = 0.263, *t* = 2.883, *p* = 0.004). Learning mode, however, does not show a significant effect (*β* = −0.025, *t* = 0.554, *p* = 0.580). Mediation analysis reveals that engagement partially mediates the relationship between support systems and motivation (*β* = 0.413, *t* = 6.066, *p* = 0.000), whereas a competitive partial mediation is observed for diverse learning resources (*β* = −0.512, *t* = 7.452, *p* = 0.000). The construct reliability and validity are confirmed with composite reliability (CR) values above 0.70, with the highest being 0.973 for learning resources, indicating strong internal consistency. These findings reinforce the Self-Determination Theory by emphasizing the role of support systems in enhancing motivation and engagement in learning, suggesting that educational institutions should focus on strengthening student support mechanisms rather than solely emphasizing learning resources or modes.

The findings of this study provide significant insights into the mediation effects of e-learning (EL) in the relationship between diverse learning resources (DLR), learning mode (LM), and support system (SS) with motivation for learning (ML), self-regulation (SR), and learning results (LR). The mediation results indicate that EL plays a crucial role in facilitating the relationships among these variables, reinforcing the conceptual framework grounded in Self-Determination Theory (SDT). The mediation effects revealed competitive partial mediation in the relationships between DLR → EL → ML and SS → EL → SR, complementary partial mediation for SS → EL → ML, and full mediation for DLR → EL → LR. These results underscore the importance of e-learning in enhancing students’ motivation, self-regulation, and academic outcomes. This pattern corresponds to the test of H7, H10, and H13, where EL functions as a mediator between DLR and ML, SR, and LR. Because the direct DLR → EL and DLR → outcome paths remain significant and in an unexpected negative direction while the indirect paths via EL are positive, these findings are interpreted as competitive partial mediation rather than full mediation within the originally hypothesized model.

The competitive partial mediation observed in the relationship between DLR, EL, and ML suggests that while diverse learning resources contribute directly to motivation, their effect is significantly enhanced when mediated through e-learning. This finding aligns with previous studies that emphasize the importance of digital learning environments in fostering student autonomy and engagement (Chiu, 2023). The role of EL as a mediator highlights the impact of well-structured online learning resources in stimulating intrinsic and extrinsic motivation, which is consistent with the principles of SDT, where autonomy-supportive environments enhance motivation (Ryan et al., 2022). Furthermore, the complementary partial mediation of SS → EL → ML suggests that while social and institutional support directly enhances motivation, the integration of e-learning platforms strengthens this effect by providing interactive learning experiences. This supports existing research asserting that digital learning platforms improve students’ psychological engagement and satisfaction, which in turn drives motivation (Merhi and Meisami, 2024).

The mediation analysis also demonstrated a full mediation effect for DLR → EL → LR, implying that the effectiveness of diverse learning resources in improving learning outcomes is entirely dependent on the presence of an e-learning platform. This is particularly relevant in blended and online learning environments, where students rely on digital materials to supplement their studies (Jehoul et al., 2025). The full mediation effect suggests that without the integration of e-learning, diverse learning resources may not translate into improved academic performance, test scores, or language proficiency. This finding aligns with research emphasizing the necessity of technology-enhanced learning environments in achieving better learning outcomes (Rafti et al., 2025). Similarly, the complementary partial mediation of SS → EL → SR highlights the crucial role of e-learning in supporting students’ self-regulatory behaviors, such as goal-setting, time management, and self-monitoring. This is in line with SDT’s argument that external support mechanisms, when channeled through digital learning environments, satisfy students’ needs for autonomy, competence, and relatedness, thereby improving their ability to self-regulate (Lin and Wang, 2025).

In contrast, the non-significant mediation results for LM → EL → ML, LM → EL → SR, and LM → EL → LR indicate that learning mode does not exert a significant influence on motivation, self-regulation, or learning results through e-learning. This finding contradicts some previous studies that suggested a direct impact of online versus traditional learning modes on academic engagement and performance (Jiang and Peng, 2025). The lack of mediation suggests that factors such as instructional quality, learner adaptability, and technological accessibility may play a more dominant role in shaping student outcomes than the mode of learning itself. This challenges the common assumption that digital learning environments automatically enhance learning effectiveness and calls for further research into the conditions under which different learning modes are most effective.

The theoretical contributions of this study are significant, as the findings extend the application of Self-Determination Theory in e-learning contexts. By demonstrating the mediating role of EL in motivation, self-regulation, and learning outcomes, this study supports the argument that digital learning environments facilitate psychological need satisfaction, thereby fostering better learning behaviors. This aligns with research by Yang Y. et al. (2025) who found that e-learning tools enhance students’ sense of autonomy and competence, which are key predictors of motivation and academic success. Moreover, the study contributes to the ongoing debate on the effectiveness of blended learning, suggesting that e-learning is not merely a substitute for traditional learning but rather a complementary mechanism that enhances the learning process.

From a practical perspective, these findings provide valuable insights for educators, instructional designers, and policymakers. The strong mediation effects of e-learning highlight the need for institutions to invest in high-quality digital learning platforms that provide diverse, interactive, and engaging resources. Furthermore, the results suggest that support systems, such as teacher guidance and peer collaboration, should be integrated into e-learning environments to enhance motivation and self-regulation. Institutions should also recognize that simply transitioning to an online learning mode is not sufficient; rather, the effectiveness of digital learning hinges on the quality of resources and support systems available to students. Future research should explore the role of additional moderating variables, such as digital literacy and student engagement, to better understand the dynamics of e-learning effectiveness.

Overall, this study provides strong empirical evidence supporting the critical role of e-learning in shaping student motivation, self-regulation, and learning outcomes. The findings align with SDT by demonstrating how digital learning environments can facilitate psychological need satisfaction, thereby enhancing learning engagement and performance. While the study confirms the mediating role of EL in most relationships, it also challenges the assumption that learning mode alone significantly influences learning outcomes. By addressing both theoretical and practical implications, this research contributes to a deeper understanding of how digital learning resources and support systems interact to shape students’ educational experiences.

This study examined the mediating role of engagement in learning within the relationships between diverse learning resources, support systems, learning modes, and motivation, using Self-Determination Theory (SDT) as its foundation. The findings confirmed that engagement is a significant mediator, underscoring the central importance of satisfying the psychological needs for autonomy, competence, and relatedness in fostering motivation. A key theoretical contribution emerging from the analysis is the proposed integration of SDT with Cognitive Load Theory (CLT), offering a more nuanced explanatory framework. This synthesis posits that even within resource-rich environments that support autonomy and relatedness, excessive intrinsic or extraneous cognitive load can overwhelm a learner’s capacity, directly undermining their sense of competence. This dual cognitive-motivational perspective compellingly explains counterintuitive results, such as the observed negative correlation between resource diversity and engagement, by suggesting that an abundance of poorly structured options can create cognitive overload, thereby negating potential motivational advantages.

Building on this integrated SDT-CLT framework, the study’s practical and methodological contributions gain greater precision. For educators and policymakers, the implication shifts from merely increasing resources to strategically designing learning ecosystems that manage cognitive load. The goal is to protect the learner’s sense of competence through clear scaffolding and coherent resource organization, thereby allowing the motivational benefits of autonomy and relatedness to fully manifest. Methodologically, the research provides a validated analytical model that now incorporates a diagnostic lens for identifying cognitive barriers to engagement, offering a more robust tool for evaluating educational interventions aimed at enhancing self-regulation and motivation.

Despite these contributions, the study acknowledges limitations, including potential sample bias and constraints on generalizability. These limitations, however, clearly chart a course for future inquiry. Subsequent research should employ longitudinal designs to trace the stability of these mediated relationships over time and explore their validity across diverse cultural contexts. Furthermore, investigating the integration of emerging digital technologies through the combined SDT-CLT lens presents a fertile ground for innovation. Future studies can directly test interventions designed to minimize extraneous cognitive load while proactively supporting psychological needs, thereby refining and applying the advanced theoretical model proposed.

## 5 Conclusion

This study used Self-Determination Theory to evaluate how involvement in learning mediates the links between varied learning resources, support systems, and learning modes and motivation for learning. Engagement strongly influences these correlations, confirming the importance of autonomy, competence, and relatedness in learning motivation. The study applies SDT to multiple learning environments and advises educators and policymakers on optimizing support structures to motivate students. A verified approach for engagement-driven motivation analysis is provided. To boost self-regulation and motivation, personalize learning resources and create interactive learning environments. Sample bias and limited generalisability to larger groups are drawbacks. Cross-cultural perspectives, longitudinal effects, and digital learning technology integration should be studied to improve these interactions.

Despite autonomy and relatedness support, intrinsic cognitive load (from complex content) or extrinsic cognitive load (from poorly designed resources) may exceed learners’ cognitive capacities, reducing their sense of competence—a core need in Self-Determination Theory. This integration may explain numerous unexpected results, such as the negative connection between resource diversity and engagement, suggesting that too many options may increase cognitive burden and reduce motivation. By explaining this connection, we may give a more advanced theoretical framework that emphasizes the dual cognitive-motivational dimensions of learning, enhancing our research’s intellectual value and practical relevance.

This study found that learning context fundamentally moderates motivation, self-regulation, and learning results. Motivation drives self-regulation, which leads to results, in traditional settings since the structured context provides scaffolding. In virtual situations, engagement fully mediates this pathway; motivation alone is insufficient without a learning design that actively promotes cognitive and behavioral involvement. Critically, the virtual environment’s capacity to promote autonomy and intrinsic motivation depends on minimizing extraneous cognitive load. Resource diversity or bad design overwhelms learners, eroding their sense of competence, interrupting self-regulation, and compromising results. Thus, traditional settings provide reliability through external structure, while virtual settings offer higher-potential, higher-risk pathways that require motivational and cognitive support to turn autonomy into productive engagement and achievement.

The study openly admits that Chinese societal and educational norms affect SDT categories. We recognize that hierarchical teacher-student dynamics and teacher-centered pedagogical approaches may affect learners’ perceptions and experiences of autonomy support, competence feedback, and relatedness differently from Western educational contexts where Self-Determination Theory (SDT) was developed. This cultural perspective is crucial for interpreting our results, especially engagement measurements, and shows that motivational frameworks may require cultural revolution. Thus, future research should focus on culturally relevant instrument development and validation, as well as cross-cultural or intra-cultural comparative studies to examine how structural factors affect SDT-based interventions and learner outcomes. This addition will make the discourse more scholarly and understandable.

This study’s focus on urban Chinese learners may introduce regional and institutional sampling bias, its cross-sectional design precludes causal or longitudinal conclusions, and its use of self-reported data may introduce measurement bias. The broad definition of “virtual learning” may also mask key variations across digital platforms and their cognitive and motivational effects. To overcome these constraints and advance the field, future research should use longitudinal and experimental designs to track causal pathways over time, integrate multi-method data like behavioral analytics to supplement self-reports, and compare the integrated SDT-CLT framework across cultures. Future studies should disaggregate virtual learning into modalities and develop focused interventions to reduce cognitive load and meet psychological requirements, turning theoretical findings into actionable educational design principles.

## Data Availability

The original contributions presented in the study are included in the article/Supplementary material, further inquiries can be directed to the corresponding author.
