# Supplementary material for: Impact of diverse learning resources and support system on Chinese English language learners’ motivation in virtual and traditional learning settings
Source: Front Psychol. 2026 Sep 7;17:1705007. doi: 10.3389/fpsyg.2026.1705007 (PMC13590768; doi:10.3389/fpsyg.2026.1705007)
Supplement: Supplementary file 1 [file Data_Sheet_4.docx]

Supplementary Material

# 1 Supplementary Data

Supplementary Material should be uploaded separately on submission. Please include any supplementary data, figures and/or tables.

Supplementary material is not typeset so please ensure that all information is clearly presented, the appropriate caption is included in the file and not in the manuscript, and that the style conforms to the rest of the article.

# 2 Supplementary Figures and Tables

Supplementary Table 5: Pangenomic analysis results

| **Category** | **Gene clusters** | **Percentage of pangenome (%)** |
| --- | --- | --- |
| Core genes | 2271 | 6.80 |
| Soft core genes | 0 | 0.00 |
| Shell genes | 11093 | 33.23 |
| Cloud genes | 20017 | 59.97 |
| Total genes | 33381 | 100.00 |

## 2.1 Supplementary Figures

**Supplementary Figure 1:** Maximum-Likelihood phylogenetic tree based on 16S rRNA gene sequences of Streptomyces strain STM-2 and closely related species within the genus Streptomyces, along with *Actinomyces oris* strain WVU474. Node numbers indicate bootstrap values, expressed as percentages from an analysis of 1000 resampled datasets. The scale bar represents 0.01 substitutions per nucleotide position.

**Supplementary Figure 2:** Maximum-parsimony phylogenetic tree based on 16S rRNA gene sequences of Streptomyces strain STM-2 and closely related species within the genus Streptomyces, along with *Actinomyces oris* strain WVU474. Node numbers indicate bootstrap values, expressed as percentages from an analysis of 1000 resampled datasets. The scale bar represents 20.00 substitutions per nucleotide position.

**Supplementary Figure 3**: Average amino acid identity (AAI) analysis tree.

**Description of gene catalogs of NRPS and PKS BGCs.**

**STM-2 encodes a wide range of non-ribosomal peptide synthases (NRPS)**

Several regions in contig 2 of the STM-2 genome were identified with NRPS genes. NPRS in region 2.1 had 23 genes with condensation, AMP-binding, EHN, MbtH, Amidohydro_2, PAD_porph, Molybdopterin, Glyco_hydro_16, Acyl-CoA_dh_1, CoA_transf_3, and Glyoxalase as biosynthetic genes; HTH_1, Rrf2, two TetR_N, MerR_1, and HTN_31 genes as regulatory genes. The MiBG comparison analysis revealed that region 2.1 was closest to xenematide-producing BGC; BGC0001825.4 (72%). No compound was identified for the KnownClusteBlast since the similarity confidence was not significant, suggesting a cryptic BGC. NRPS in region 2.4 also had 38 genes with PIG-L, two adh_short_C2, Acyl-CoA_dh_N, ECH_1, LCM, Acettyltrasnf_3, Carbam_trans_N, two Epimerase, AMP-binding, Atu4866, Gp_dh_N, Abdhydrolase_3, methyltransf_11, Glyoxylase and NAD_binding_2 genes as biosynthetic genes; TetR_N, HTH_31, HTH_18, HTH_3 and MarR genes as regulatory genes, and two MFS_1 genes as transport-related genes. Based on the MiBG comparison, NRPS BGC in region 2.4 was closest to the BGC encoding atromentin (65%) produced by *Suillus grevillei*. There were 36 genes identified in the NRPS region 2.7, with two FGGY_N, SseB, BacA, Oxidored_FMN, adh_short, DAO, Cupin_2, adh_short, Cu_amine_oxid, AMP-binding, Condensation, MbtH, Methyltransf_19, p450, Glyco_hydro_20b, and Gyco_hydro_28 as biosynthetic genes; three TetR_N genes, HTH_31, HTH_3, and Sigma70_r2/r4_2 genes as regulatory genes, and MFS_1 gene as transport-related gene. The MiBG comparison revealed that NRPS in region 2.7 was closest to *Xenorhabdus bovienii* SS-2004’s BGC that encodes bovienimide A (75%). NRPS-like-terpene-precursor BGC with 56 genes was identified in region 2.9 of the STM-2 genome. The biosynthetic genes were NMT1, Sulfatase, Glyco_hydro_2, FGE_sulfatase, Aldolase_II, Bac_luciferase, Condensation, AMP-binding, Aminitran_1_2, Methyltransf_11, GFO_IDH_MocA, AP_endonuc_2, FAD_binding_3, Glyco_hydro_cc, HD, FAA_hydrolase, MR_MLE_C, polyprenyl_synt, Acetyltransf_3, Tautomerase_2, BPL_LpIA_LipB, and Putative_PNOx. Two Lacl, HTH_24, two TetR_N, Response_reg, GntR, and HisKA_3 genes were identified as regulatory genes, and three BPD_transp_1, ABC_tran, and MFS_1 as transport-related genes.

NRPS in region 2.23 also had 36 genes with ADH_N, FAD_binding_4, Glyoxalase, Lon_C, ResIII, CDO_I, Adhydrolase_1, Condensation-AMP-binding, MoCoBD_1, Fer2_2, FAD_binding_5, Glyco_hydro_20, DUF3039, EP SP_synthase, and malic-Malic_M genes as biosynthetic genes; HTH_31, HTH_24, HTH_IcIR and HTH_1 as regulatory-related genes and two BBP_transp_1 and MFS_1 genes as transport genes. MiBG comparison revealed that region 2.23 was closest to BGC0002075.3 (74 %), which synthesizes pyreudione A, pyreudione B, pyreudione C, pyreudione D, and pyreudione E from *Pseudomonas fluorescens* genome. Additionally, the KnownClusterBlast module revealed a medium similarity score with ochronotic pigment biosynthetic gene cluster (BGC0000918) from *Streptomyces avermitilis.* There was an NRPS-arylpolyene gene cluster in region 2.25 of the STM-2 genome. The region consisted of 63 genes with Glyco_transf_20, Trehalose_PPase, DUF3263, Amidohydro_1, PfkB, two Flavin_Reduct, Uma2, ADH_N, Peptidase_M4, three Condensation-AMP-binding-PP-binding, three p450, Bac_luciferase, Glyoxalase, FMN_dh, PDH_N, MbtH, DSBA, adh_short_C2, two FabA, three ketoacyl-synt, PP-binding, Thioesterase, NTP_transferase, Doxx, SpoU_sub_bind, tRNA-synt_1e-DALR_2, YgbB and IspD as biosynthetic genes; TetR_N, Trans_reg_C, HTH_IcIR-IcIR, HTH_1 genes as regulatory genes and two ABC_tran and ABC2_membrane genes as transport-related genes. Based on the MiBG comparison analysis, NRPS region 2.25 showed the highest similarity to BGC0001758.4 (45%) from Paraburkholderia rhizoxinica HKI 454, which encodes rhizomide A, rhizomide B, and rhizomide C. The KnownClusterBlast analysis revealed that region 2.25 is moderately similar to BGC0000429, the skyllamycin A/B biosynthetic gene cluster from *Streptomyces* sp. Acta 2897. NRPS-like was observed in region 2.31 of the STM-2 genome. The region 2.31 consisted of 37 genes, including Peptidase_M7, Putative_PNPOx, Sod_Ni, zf-CGNR, Methyltransf_25, malic-Malic_M, ABM, AMP-binding, Epimerase, Nitroreductase, adh_short_C2, 2-oxoacid_dh genes as biosynthetic genes; HTH_1, TetR_N, ABATE, PadR, TetR_N-TetR_C_7, Trans_reg_C and MerR_1 genes as regulatory genes and ABC_tran, BPD_transp_1 genes as transport genes. It was observed that the region 2.31 was similar to echoside A encoding BGC0000340.5 (86%) from *Streptomyces* sp. LZ35 based on the MiBG comparison analysis results.

Additionally, there were NRPS observed in regions 2.37, 2.40, 2.48, 2.49, and 2.50. NRPS region 2.37 had 41 genes, including FAD_binding_3, MDMPI_N, TauD, two p450, two Condensation-AMP-binding-AMP-binding_C-PP-binding, Abhydrolase_6, Glyco_hydro_18_Polyketide_cyc2, Metallophos, Pkinase, and Aldedh genes as biosynthetic genes; TetR_N, HTH_34, and TetR_N-TetR_C_33 genes as regulatory-related genes, and ABC2_membrane and ABC_tran genes as transport genes. Based on the MiBG comparison analysis, region 2.37 showed 74% similarity to BGC from *Burkholderia gladioli* (BGC0001833), which encodes icosalide. NRPS region 2.40 consisted of 35 genes, including Glyco_hydro_43, Glyco_hydro_2_N, HATPase_c_2, Methyltransf_19, LacAB_rpiB, AMP-binding, FGGY_N-FGGY_C, FAD_binding_2, five adh_short_C2, two APH, three Acyl-CoA_dh_N-Acyl-CoA_dh_M-Acyl-CoA_dh_1, Acyl-CoA_dh_N-Acyl-CoA_dh_1, ECH_1, Thiolase_N-Thiolase_C genes as biosynthetic genes; HTH_31, Lacl and Response_reg as regulatory genes and Sugar_tar-MFS_1 as transport-related gene. It was observed that region 2.40 was 53% similar to the choline biosynthetic gene cluster (BGC0002276) from *Aspergillus nidulans* FGSC A4 based on the MiBG comparison analysis results. It was observed that region 2.48 had 36 genes, including Rieske-Aromatic_hydrox, Abhydrolase_1, two CoA_transf_3, AMP-binding-AMP-binding_C, Abhydrolase_6, Cupin_2, Wzy_C, Amidohydro_1, AMP-binding-AMP_binding_C-PP-binding-Condensation, MbtH, Abhydrolase_6, two OCD_Mu_crystall, SNARE_assoc, Acyl-CoA_dh_N-Acyl-CoA_dh_M-Acyl-CoA_dh_1, p450, Aminotran_1_2, DHDPS, FAA_hydrolase genes as biosynthetic genes; HTH_AsnC-type, Response_reg-GerE, HisKA_3, TertN, and HTH_31 as regulatory-related genes. MiBG comparison analysis revealed that region 2.48 was 74% similar to pyreudione A biosynthetic gene cluster (BGC0002075) from *Pseudomonas fluorescens.* KnownClusterBlast analysis also revealed low similarity to glycinocin A biosynthetic genBGC0000379e cluster from *Streptomyces viridochromogenes,* suggesting that region 2.48 could potentially synthesize a novel compound. NRPS-like region 2.49 had 38 genes consisting of 17 biosynthetic genes, 3 regulatory genes, 3 transport genes, and 15 other genes. The biosynthetic genes of region 2.49 included Peptidase_S15-PepX_C, Carbam_trans_N-Carbam_trans_C, Glycos_transf_2, Methyltransf_11, GARS_A, PIGL-L, Epimerase, GFO_IDH_MocA-GFO_IDH_MocA_C, TauD, AMP-binding-AMP-binding_C-PP-binding, Aminotran_1_2, APH, Biotin_carb_N-CPSase_L_D2-Biotin_carb_C-Biotin_lipoyl, ACC_epsilon, Carboxyl_trans, AXE1, and Glyco_hydro_127 genes. HTH_IcIR, TetR_N, and Lacl-Peripla_BP_3 genes were identified as regulatory genes, and ABC_membrane, ABC_tran, and BPD_transp_1 as transport genes. Based on the MiBG comparison analysis, NRPS region 2.49 showed the highest similarity to BGC0001379.5 (71%) from *Streptomyces luteocolor*, which encodes BD-12. Region 2.50 was a hybrid of NRP-metallophore-NRPS-T1PKS, which had 62 genes, including AMP-binding-AMP-binding_C, Ald_Xan_dh_C_MoCoBD_1-MoCoBD_2, ECH_1, FAD_binding_5-CO_deh_flav_C, Fer2-Fer2_2-COXG, Asn_synthase, AIRC, two AP_endonuc_2, GFO_IDH_MocA-GFO_IDH_MocA_C, Formyl_trans_N-Formyl_trans_C, Lys_Orn_oxgnase, AMP-binding-AMP_binding_C-PP-binding-Condensation, Esterase, Amidohydro_3, FAD_binding_3, two Ketoacyl-synt-Ketoacyl-synt_C-Acyl_transf_1-PP-binding, two Ketoacyl-synt-Ketoacyl-synt_C-Acyl_transf_1-KR-PP-binding, four Docking-Ketoacyl-synt-Ketoacyl-synt_C-Acyl_transf_1-KR-PP-binding, Esterase_PHB, and Tannase-3HBOH genes as biosynthetic genes; PadR, two LacL, HTH_1, HTH_IcIR-IcIRHTH_18 genes as regulatory genes and ABC_tran and two ABC_membrane genes as transport genes. Based on the MiBG comparison analysis, region 2.50 showed very high similarity (138%) to the 6-methylsalicylic acid biosynthetic gene cluster (BGC0001275) from *Glarea lozoyensis.*

**STM-2 encodes a wide range of polyketide synthases (PKS)**

The antiSMASH analysis revealed 13 regions in the genomes of STM-2 that encode polyketide synthases. PKS-like-terpene hybrid region 2.5 had 37 genes with Haem_oxygenas_2, Fer2-Fer2_2, FAD_binding_5-CO_deh_flav_C, Ald_Xan_dh_C-MoCoBD_1, Methyltransf_11, PP-binding, Rieske, two p450, ACP_syn_III_C, adh_short_C2-ketoacyl-synt-ketoacyl-synt_2-KR, Acyl_transf_1-PP-binding, Abhydrolase_1, two ADH_N-ADH_zinc_N, SQS_PSY, MDMPI_N and Endonuclease_1 as biosynthetic genes; two TetR_N genes as regulatory genes and two MFS_1 genes as transport-related genes. Based on the MiBG comparison analysis, PKS-like-terpene hybrid region 2.5 showed the highest similarity to BGC0001948.3 (48%) from *Streptomyces* sp., which encodes naseseazine C, C3-aryl pyrroloindolines. The knownClusterBlast analysis also revealed closest similarity to rustimicin biosynthetic gene cluster from *Streptomyces galbus* with low similarity confidence. This indicates region 2.5 has the potential of synthesizing novel or different forms of antifungal compound. There were 46 genes in T1PKS-NRPS-like hybrid region 2.10 including DHquinase_II, DegT_DnrJ_EryC1, GFO_IDH_MocA-GFO_IDH_MocA_C, PepSY_TM, Pyr_redox_2, Trypsin, p450, Bac_liciferase_Acetyltrans_2, two Ketoacyl-synt-Ketoacyl-synt_C-Acyl_transf_1-KR-PP-binding, two Docking-Ketoacyl-synt-Ketoacyl-synt_C-Acyl_transf_1-KR-PP-binding, AMP-binding, two FAD_binding_3, Nitroreductase, ADH_N-ADH_zinc_N, FMO-like, Sdh_cyt, and FAD_binding_2 as biosynthetic genes; PadR, sigma70_r2-sigma70_r4_2, TetR_N and MarR as regulatory genes. It was observed that the region 2.10 was 91% similar to the hygrocin A/hygrocin B biosynthetic gene cluster (BGC0000075) from *Streptomyces* sp. LZ35 based on the MiBG comparison analysis results.

Another T1PKS-NRPS hybrid cluster was observed in region 2.11 of STM-2 genome, which had 52 genes in total. The genes consisted of Methyltransf_11, Glyco_hydro_127, Epimerase, Putative_pNPOx, p450, two Docking-Ketoacyl-synt-Ketoacyl-synt_C-Acyl_transf_1-KR-PP-binding, Ketoacyl-synt-Ketoacyl-synt_C-Acyl_transf_1-PP-binding, Condesation-AMP-binding-PP-binding, Alpha-amylase, arginosuc_synth and Glyco_hydro_62 as biosynthetic genes; two TetR_N, MerR_1, Response_reg-GerE, HisKA_3, two HTH_1, and Lacl genes as regulatory genes and three MFS_1 and two BPD_transp_1 genes as transport-related genes. The MIBiG comparison analysis revealed 77% similarity to meridamycin BGC (BGC0001012.3) from *Streptomyces violaceusniger.* Region 2.18 was a hybrid ofT1PKS-NI-siderophore hybrid, which had 44 genes in total, including polysacc_deac_1, ADI, Methyltransf_11-NUDIX, Methyltransf_11, FAD_binding_3, Ketoacyl-synt-Ketoacyl-synt_C-Acyl_transf_1-PP-binding, CMD, Methyltransf_3, Acyl-CoA_dh_N-Acyl-CoA_dh_M-Acyl-CoA_dh_1, PP-binding, 3HCDH_N-3HCdh, GATase, Uma2, Glyoxalase, Orn_Arg_deC_N, two IuCA_IucC, ATPgrasp_N-ATP-grasp_4, Carboxyl_trans, LPMO_10, Lipase_2, IMS_C and Putative_PNPOx as biosynthetic genes; GntR, TetR_N, Response_reg-GerE, HisKA_3, HTH_18 and MarR as regulatory genes and MFS_1 as transport gene. Based on the MIBiG comparison analysis, the region was 56% similar (closest) to xanthoferrin biosynthetic gene cluster (BGC0001408) from *Xanthomonas oryzae pv. oryzae* KACC 10331. The knownClusterBlast results showed a very low significant similarity to peucechelin biosynthetic gene cluster from *Streptomyces peucetius* subsp. *caesius* ATCC 27952, suggesting the potential for the region to produce novel or different forms of these similar compounds. T1PKS cluster was observed in region 2.20 of STM-2 genome. The region 2.20 had 56 genes in total, which included Amino_oxidase, AMP-binding, two Thioesterase, three Docking-Ketoacyl-synt-Ketoacyl-synt_C-Acyl_transf_1-KR-PP-binding, three Docking-Ketoacyl-synt-Ketoacyl-synt_C-Acyl_transf_1-PP-binding, PP-binding-Ketoacyl-synt-Ketoacyl-synt_C-Acyl_transf_1, Aldedh, TPP_enzyme_N-TPP_enzyme_M-TPP_enzyme_C and Kdul as biosynthetic genes; Response_reg-GerE, and HisKA_3-HATPase_c genes as regulatory genes and ABC_tran and ABC2_membrane as transport-related genes. The region was observed to be 82% similar to 6-methylsalicyclic acid biosynthetic gene cluster (BGC0001275) from *Glarea lozoyensis* based on MIBiG comparison analysis. According to the knownClusterBlast analysis, the region showed medium similarity with desulfoclethramycin biosynthetic gene cluster (BGC0002498) from *Streptomyces* sp. Region 2.22 of STM-2 genome was identified as HR-T2PKS hybrid with a total of 55 genes. Genes involved in biosynthesis I region 2.22 were Amidase, Abhydrolase_6, ACPS, Biotin_Carb_N-CPSase_L_D2_Biotin_carb_C-Biotin_lipoyl, DSBA, two adh_short_C2, FabA, two PP-binding, Acetyltransf_1, two Ketoacyl-synt, two Ketoacyl-synt-Ketoacyl-synt_C, ADH_n-ADH_zinc_N, Bac_luciferase, NMT1, LPMO_10 and PG_binding_1 genes. The region 2.22 also includes HTH_IcIR-IcIR, GerE and HTH_31 as regulatory genes and three BPD_transp_1, ABC_tran and two MFS_1 genes as transport-related genes. The genes involved in HR-T2PKS region 2.22 were closest in similarity (64%) to those genes involved in the synthesis of 6-methylsalicyclic acid from *Aspergillus terreus* based on the MIBiG comparison analysis. The knownClusterBlast analysis also revealed that HR-T2PKS region 2.22 was medium closest in similarity to (2E, 4E, 6E, 8E)-N-(2-hydroxy-5-oxocyclopent-1-en-1-yl)-9-(o-tolyl)nona-2,4,6,8-tetraenamide biosynthetic gene cluster (BGC0002441) from *Streptomyces* sp. SANK 60404,

Additionally, there were PKS clusters in regions 2.34, 2.39, 2.44, 2.45, 2.46, 2.47 and 2.52 of STM-2 genome. The T2PKS region 2.34 consisted 67 genes in total, including two Methyltransf_11, ACPS, Acetyltransf_3, DUF2795, Phage_holi_3_6, MDMPI_N, catalase-catalase-rel, TPP_enzyme_N-TPP_enzyme_M-TPP_enzyme_C, Bac_luciferase, Aminotran_1_2, Methyltransf_2, Cyclase_polyket, polyketide_cyc, PP-binding, two Ketoacyl-synt-Ketoacyl-synt_C, Cupin_2, ABm-SchA_CurD, FAD_binding_3, SNARE_assoc, Peptidase_M19, Abhydrolase_6, FAD_binding_3, Acetyltransf_3, PAS_3-HATPase_c_2, OKR_DC_1_N, PCMT, Phage_holin_4_2-Phosphodiest and PEP_mutase as biosynthetic genes; MarR_2 and TetR_N as regulatory genes and ABC_tran and BPD_transp_2 as transport genes. Based on MIBiG comparison analysis by AntiSMASH, the genes involved in T2PKS region 2.34 were closest in similarity (76%) to those genes involved in the spore pigment production (BGC0000271.5) by *Streptomyces avermitilis.* A total of 31 genes were involved in T1PKS region 2.39. The T1PKS region 2.39 consisted of Methyltransf_19, WS_DGAT_C, Ketoacyl-synt-Ketoacyl-synt_C-Acyl_transf_1-Methyltransf_12-KR-PP-binding, AurF, Mur_ligase_M and peroxidase genes as biosynthetic genes; HTH_3, TetR_N, FUR, and Sigma70_r2-Sigma70_r4_2 as regulatory-related genes. The MIBiG comparison analysis showed that T1PKS region 2.39 cluster are in closest similarity (62%) to prolipyrone B biosynthetic gene cluster (BGC0002191) from *Fusarium graminearum* PH-1.

Genes involved in the region T1PKS 2.44 are in closest similarity (87%) to efomycin K biosynthetic gene cluster (BGC0002291) from *Streptomyces* sp. M56 based on the MIBiG comparison analysis. Efomycin K is known to have antifungal properties and antibacterial activity. A total of 39 genes were identified in the T1PkS 2.44 region, including CobN-Mg-chei, ACP_syn_III-ACP_syn_III_C, 3HCDH-N-3HCDH, NTp_transferase, two Epimerase, Ketoacyl-synt-Ketoacyl-synt_C-Acyl_transf_1-PP-binding, three Docking-Ketoacyl-synt-Ketoacyl-synt_C-Acyl_transf_1-KR-PP-binding, Ketoacyl-synt-Ketoacyl-synt_C-Acyl_transf_1-KR-PP-binding, Thioesterase, DUF1205-UDPGT, dTDP_sugar_isom, Aldo_ket_red, hexose_dehydrat, ADH_zinc_N, Fapy_DNA_glyco-H2TH, FAD_binding_3, and pyr_redox_2 as biosynthetic genes; Response_reg-GerE, and HisKA_3-HATPase_c genes as regulatory genes and ABC-tran and ABC2_membrane genes as transport genes. T1PKS region 2.45 had 36 genes in total, including Anmk, CMD, Aminotran_4, AMP-binding-FAD_binding_3, GATase-Anth_synt_I_N-Chorismate_bind, Aminotran_3, Ketoacyl-synt-Ketoacyl-synt_C, Ketoacyl-synt-Ketoacyl-synt_C-Acyl_transf_1-KR-PP-binding, GFO_IDH_MocA-GFO_IDH_MocA_C, Cupin_2, adh_short_C2, Kdo, Thioesterase, PfkB, Indigoidine_A, PhzC-PhzF and YjbR as biosynthetic genes; HTH_AsnC-type, HTH_18, TetR and HTH_24 as regulatory genes. Genes contained in T1PKS region 2.45 were observed to be in closest similarity (65%) to PKS gene cluster (BGC0001273.3) involved in the synthesis of asperlactone from *Aspergillus ochraceus,* according to the MIBiG comparison analysis.

There were 61 genes identified in T1PKS region 2.46, including Acetyl_transf_1, Aldedh, Pyr_redox_2, AAA_2, PGA_cap, LCM, two Peptidase_S15, Methyltransf_11, Ketoacyl-synt-Ketoacyl-synt_C-Acyl_transf_1-PP-binding, Nine Docking-Ketoacyl-synt-Ketoacyl-synt_C-Acyl_transf_1-KR-PP-binding, Abhydrolase_1, p450, PP-binding, AMP-binding, ArsC, Peptidase_M48, and FAD_binding_3 as biosynthetic genes; Sigma70_r2-Sigma70_r4_2, Trans_reg_C, and Penicillinase_R as regulatory genes and ABC2_menmbrane_2 as transport-related genes. Based on the MIBiG comparison, the genes in T1PKS region 2.46 showed the closest similarity (88%) to genes involved in the synthesis of nigericin (BGC0000114.5) from *Streptomyces violaceusniger.* The T1PKS region 2.47 had a total of 68 genes, including ADH_N, Putative_PNPOx, DUF3516, YdjM, YkuD, p450, Fer4_19, seven Docking-Ketoacyl-synt-Ketoacyl-synt_C-Acyl_transf_1-KR-PP-binding, PP-binding-Ketoacyl-synt-Ketoacyl-synt_C-Acyl_transf_1-KR-PP-binding, AMP-binding, Abhydrolase_1, Cupin_2, Methyltransf_11, CoA-transf_3 and CN_hydrolase genes as biosynthetic genes; MarR_2, two TetR_N, HTH_3, GntR-FCD and HTH_18 genes as regulatory genes and three ABC_membrane genes as transport-related genes. Genes contained in T1PKS region 2.47 were observed to be in closest similarity (86%) to PKS gene cluster (BGC0001700.5) involved in the synthesis of niphimycin C, niphimycin D, niphimycin E, 17-O-methylniphimycin, niphimycin Iα, and 19-O-malonylniphimycin from *Streptomyces* sp. IMB7-145 based on the MIBiG comparison analysis. Niphimycin and its related isoforms are known as antimicrobial compounds with antifungal activity. A total of 40 genes were observed in the T3PKS region 2.52. The genes region 2.52 consisted three HATPase_c_2, zf-CGNR, Aminotran_1_2, Glyoxalase, ADH_N-ADH_zinc_N, Chal_sti_synt_N, two RibD_C, FA_desaturase, ADH_zinc_N and F420_oxidored genes as biosynthetic genes; ABATE, two HTH_1, three TetR_N genes as regulatory genes. MiBG comparison analysis revealed that region 2.48 was 86% similar to germicidin (antimicrobial agent with antifungal activity) biosynthetic gene cluster (BGC0001454.5) from *Streptomyces argillaceus,* suggesting the potential of STM-2 synthesizing germicidin.
